# Can CMR be the new ‘gold standard’ for constrictive pericarditis?

**DOI:** 10.1186/1532-429X-14-S1-O33

**Published:** 2012-02-01

**Authors:** John A Power, Diane A Vido, Mark Doyle, Robert W Biederman

**Affiliations:** 1 University of Rochester, Rochester, NY, USA; 2 Allegheny General Hospital, Pittsburgh, USA

## Summary

Historically, the cardiac catheterization has been the 'gold standard'for the evaluation and confirmation of constrictive pericarditis. Noninvasive CMR should have a potential role to be equivalent or even superior given its abiltiy to detect both the anatomic and physiologic impact of an abnormal pericardium. Via RF tissue-tagging, we show that CMR could be a contender for a new non-invasive standard for the evalaution of this often clinical conundrum.

## Background

Perhaps no cardiac disorder is as elusive as constrictive pericarditis (CP) mimicking diseases as divergent as MI to cirrhosis. The penalty for incorrect decision is more dire; needless sternotomy vs. continued undiagnosed symptoms. Classically, invasive cardiac catheterization (cath) has been thought to be the *defacto* ‘gold standard’ and more recently echocardiography has shown promise as a non-invasive modality. However, sensitivity and specificity for both vary dramatically from center to center with either suffering from false positives. Given the ability to unequivocally define large segments of the pericardium and to define its physiologic interaction with the heart via radio-frequency (RF)tissue tagging, CMR may have a unique capability to be a new ‘gold standard’. We hypothesize that CMR via SSFP and RF tissue tagging can define visceral-parietal adherence and achieve a high degree of accuracy as compared to surgically proven CP.

## Methods

All patients referred for CMR evaluation of CP underwent a retrospective review of CMR RF tagging accuracy as compared to inter-operative surgical findings. Similarly, all ancillary testing pre-OR was reviewed in a blinded manner.

All records and CMR examinations from Jan 2000-Aug 2011 were reviewed and collated representing 118 consecutive pts.

## Results

One-hundred eighteen (118) pts were referred for clinical suspicion of CP. Thirty-seven(37) pts were defined as CP+ via CMR from which 21/37 (57%) were cath-defined CP+, 4 CP- while 1 was equivocal. (In 55% the cath followed CMR). Of these, 16/27 (59%) were surgically confirmed. There was 100% agreement between CMR and surgical findings. The remainder of those CP+ patients (10) were treated medically, declined or were ineligible for surgery or were lost to follow-up. Eighty-one (81) pts were defined CP- via CMR from which 18/24 (75%) underwent cath and were cath-defined CP-, 5 were CP+ while 1 was CP equivical. Of these, only 2/81(2%) went to surgery and neither had CP (one pt had an intrapericardial mass as demonstrated on CMR). In those defined as CMR CP+, only 12 underwent echo for CP suspicion with 7/12 (58%) eventually shown to be surgically-proven CP+. Overall, in the pts that underwent surgery, there was 100% agreement between CMR and the surgical findings. The agreement rate was similar between cath and surgical findings.

## Conclusions

CMR via RF tissue-tagging offers an unique manner of defining clinically relevant constrictive pericarditis as confirmed at surgery. Specifically, no pt identified as CP+ underwent inappropriate pericardiectomy, nor was any CMR CP- pt ever shown to be CP+. Against catheterization, with or without echocardiography, there were substantial and unacceptable false positives and false negatives. Limiting complete validation is the understood inability to surgically prove those CMR CP- patients. Nevertheless, we propose that CMR, upon further validation, has the characteristics to be the new ‘reference standard' for constrictive pericarditis.

## Funding

Internal.

